# Advancing future research on sepsis outcomes in adolescents with SUD: integrating AI, accounting for temporal trends, and enhancing exposure classification

**DOI:** 10.1186/s13613-025-01556-7

**Published:** 2025-09-05

**Authors:** Havell Markus, Gary D. Ceneviva, Neal J. Thomas, Conrad Krawiec

**Affiliations:** 1 Pennsylvania State University College of Medicine, 500 University Drive, P.O. Box 850, Hershey, PA 17033-0850 USA; 2 Program in Bioinformatics and Genomics, Pennsylvania State University, Huck Life Sciences Building, PA 16802 University Park, USA; 3 Medical Scientist Training Program, Pennsylvania State University College of Medicine, 500 University Drive, Hershey, PA 17033 USA; 4 Pediatric Critical Care Medicine, Department of Pediatrics, Penn State Hershey Children’s Hospital, 500 University Drive, P.O. Box 850, Hershey, PA 17033-0850 USA; 5 Department of Public Health Sciences, College of Medicine, Pennsylvania State University, 500 University Drive, Hershey, PA 17033-0850 USA

We would like to sincerely thank all the authors for their thoughtful and constructive comments on our recent study examining the impact of substance use disorders (SUDs) on critical care outcomes in adolescents with sepsis. We appreciate their engagement with our work and the opportunity to respond.

Several important themes were raised that merit discussion

1. **Opportunities for artificial intelligence integration**

The comments by Yu [1] highlight a meaningful opportunity to leverage artificial intelligence **(**AI) powered tools to help address the complex challenge of identifying and managing SUDs in adolescents. These conditions are often complex and frequently overlooked in standard clinical practice.

We agree that by training AI on a wide range of data—including medical records, social determinants of health, and behavioral patterns—we can automate the assessment of SUD risk [2]. This approach may facilitate earlier identification of at-risk adolescents, enabling more timely and effective interventions. Moreover, predictive models hold promise for stratifying risk among adolescents with SUDs, paying the way for more tailored and proactive care. Ultimately, AI has the potential to transform the identification and treatment of adolescents with SUDs, leading to more effective and individualized care – and improved long-term outcomes.

2. **Temporal shifts in sepsis and SUD definitions**

We appreciate the thoughtful comments by Zhuo [3] regarding the potential confounding impact of evolving sepsis definitions and coding practices, and we agree this is a critical factor to consider in interpreting our findings. The transition from SIRS-based definitions to organ dysfunction-based Sepsis-3 criteria in 2016 likely contributed to definitional drift in ICD-10 coding across our 2008–2023 dataset [4]. As a result, earlier cases may reflect a broader definition of sepsis than later ones, complicating direct comparisons of disease severity over time.

Crucially, SUD definitions and coding have also evolved during our study period. Policy changes such as the legalization of cannabis in many U.S. states beginning around 2012, along with growing public recognition of SUDs as medical conditions, likely influenced both documentation and screening practices [5]. Thus, if SUD diagnoses have become more comprehensive in recent years, our analysis may underestimate the association between SUD and high resource utilization, as earlier cases may have been misclassified or excluded from the exposed cohort.

We acknowledge that our current analysis may not fully account for this definitional drift in both sepsis and SUDs. This highlights a critical need for future studies to investigate how these evolving definitions and coding practices influence observed trends in sepsis incidence, severity, and outcomes, especially in vulnerable populations like those with SUDs. Such research will ultimately enhance the robustness of longitudinal data in this important public health area.

3. **Additional considerations for confounding and exposure classification**

We appreciate the comment by Yang et al. [6] regarding all-cause mortality, infection source, and the binary treatment of SUD. These observations underscore the value of more granular data to enhance risk adjustment and provide deeper insight into the impact of SUDs.

Relying on all-cause mortality limits specificity, as it does not distinguish deaths directly attributable to sepsis or SUD. Access to cause-specific mortality data would enhance interpretability. Additionally, our analysis did not adjust for infection source—a key determinant of sepsis trajectory [4]—which may confound the observed associations with SUD. Incorporating more granular data on infection sites or microbiological findings would help address this limitation by allowing for more precise risk adjustment and a better understanding of how pathogen type and infection source interact with SUD to influence outcomes. Lastly, treating SUD as a binary variable overlooks important clinical heterogeneity, such as substance type and severity [7]. More detailed exposure data would enable analysis of dose-response relationships and differential impacts across substances.

While these limitations stem from current data constraints, they also outline a clear roadmap for future research. Access to more detailed clinical data would allow for refined risk adjustment and a deeper understanding of the complex interplay between SUD and sepsis outcomes.

In summary, we appreciate the thoughtful suggestions provided by the authors and agree that many of these considerations highlight valuable directions for future research as the field continues to evolve.
